# Drug Advances in NAFLD: Individual and Combination Treatment Strategies of Natural Products and Small-Synthetic-Molecule Drugs

**DOI:** 10.3390/biom15010140

**Published:** 2025-01-17

**Authors:** Xing Wan, Jingyuan Ma, He Bai, Xuyang Hu, Yanna Ma, Mingjian Zhao, Jifeng Liu, Zhijun Duan

**Affiliations:** 1 The First Affiliated Hospital of Dalian Medical University, Dalian 116012, China; wanx03@dmu.edu.cn (X.W.); baih01@dmu.edu.cn (H.B.); zhaomj@dmu.edu.cn (M.Z.); 2 Institute of Integrated Traditional Chinese and Western Medicine, Dalian Medical University, Dalian 116051, China; 3 The First Clinical Medical College, Liaoning University of Traditional Chinese Medicine, Shenyang 110033, China; majingyuan774@gmail.com (J.M.); 98yanama@gmail.com (Y.M.); 4 The Second Clinical Medical College, Liaoning University of Traditional Chinese Medicine, Shenyang 110033, China; hux227762@gmail.com

**Keywords:** NAFLD, natural products, small-synthetic-molecule drugs

## Abstract

Non-alcoholic fatty liver disease (NAFLD) has become the most common chronic liver disease and is closely associated with metabolic diseases such as obesity, type 2 diabetes mellitus (T2DM), and metabolic syndrome. However, effective treatment strategies for NAFLD are still lacking. In recent years, progress has been made in understanding the pathogenesis of NAFLD, identifying multiple therapeutic targets and providing new directions for drug development. This review summarizes the recent advances in the treatment of NAFLD, focusing on the mechanisms of action of natural products, small-synthetic-molecule drugs, and combination therapy strategies. This review aims to provide new insights and strategies in treating NAFLD.

## 1. Introduction

**Figure 1:**
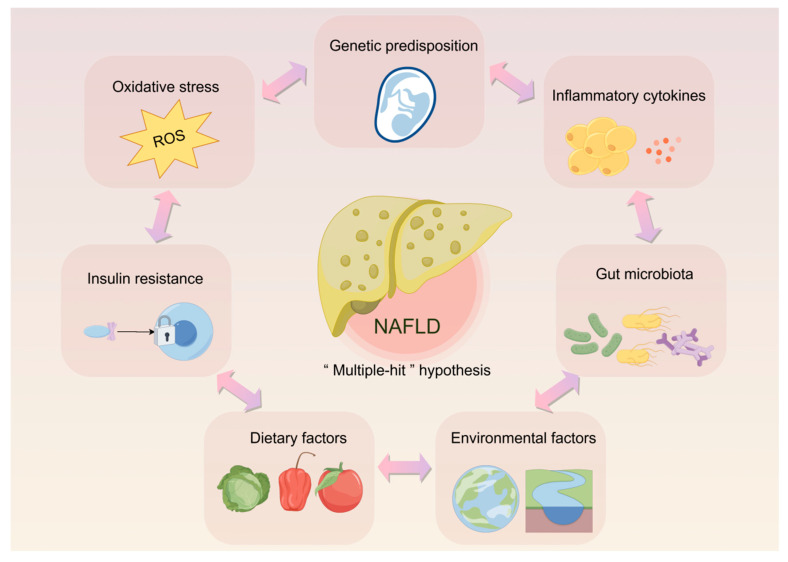
The “multiple-hit” hypothesis of NAFLD. By Figdraw.

Non-alcoholic fatty liver disease (NAFLD) is a spectrum of disorders characterized by an excessive accumulation of fat in the liver, excluding the causes of alcohol consumption or other specific damaging factors [1]. The spectrum includes simple steatosis (SS) and non-alcoholic steatohepatitis (NASH), which can progress to cirrhosis and liver cancer [2]. Recently, the terminology for NAFLD has been updated to metabolic-dysfunction-associated fatty liver disease (MASLD), reflecting its close association with metabolic dysregulation. Similarly, NASH has been redefined as metabolic-dysfunction-associated steatohepatitis (MASH) [3]. Increasing evidence suggests that the “two-hit” hypothesis is no longer sufficient to explain the complex pathogenesis of NAFLD, hence the emergence of the “multiple-hit” hypothesis [4]. This framework emphasizes the interaction of various factors in the development and progression of NAFLD, including insulin resistance, inflammatory cytokines, oxidative stress, gut microbiota dysbiosis, genetic susceptibility, and environmental and dietary influences (see Figure 1) [5]. A recent meta-analysis estimated that NAFLD affects more than 30% of adults worldwide, with a higher prevalence in patients with obesity, type 2 diabetes mellitus (T2DM), and metabolic syndrome [6]. Prevalence rates vary regionally, with the highest rates reported in Latin America (44.37%), Middle East and North Africa (36.53%), South Asia (33.83%), Southeast Asia (33.07%), North America (31.20%), and Western Europe (25.10%) [6]. NAFLD is projected to become the major cause of liver cancer, suggesting that effective interventions are urgently needed to mitigate its impact on global health [7].

Despite the high prevalence and serious complications of NAFLD, effective treatment options remain limited. Current clinical management primarily relies on lifestyle interventions, including weight loss, dietary modifications, and increased physical activity. However, these approaches are often hindered by a poor adherence and the limited efficacy in reversing advanced stages of the disease [8]. Pharmacological therapies have shown promise in improving certain aspects of NAFLD [9,10,11]. Natural products, widely used in traditional medicine, have received increasing attention for their potential therapeutic effects in NAFLD [12,13,14]. Many natural compounds possess antioxidant, anti-inflammatory, and metabolism-regulating properties that may attenuate hepatic steatosis and reduce the risk of inflammation and fibrosis [15,16,17]. In addition, the multicomponent nature of natural products may produce synergistic therapeutic effects [18]. Meanwhile, small-synthetic-molecule drugs have emerged as a promising area of research for the targeted therapy of NAFLD. These drugs aim to modulate lipid metabolism, reduce hepatic inflammation, prevent hepatic fibrosis, and improve insulin sensitivity with considerable therapeutic potential [19]. This review comprehensively summarizes recent advances in the study of natural products and small-synthetic-molecule drugs for the treatment of NAFLD. Furthermore, it explores the potential of combination therapeutic strategies, providing new perspectives and approaches for the precise treatment of NAFLD.

## 2. Retrieval Strategy

To provide a comprehensive and up-to-date review of therapeutic advances for NAFLD, we conducted a systematic search of the literature using the PubMed and Web of Science databases. The search covered studies published from January 2010 to November 2024. The following keywords were used in the search: “nonalcoholic fatty liver disease” OR “metabolic dysfunction associated fatty liver disease” AND “natural products” OR “small molecule drugs” AND “pathogenesis” OR “treatment” OR “therapeutics”. The searches were limited to English-language publications involving human or animal studies relevant to NAFLD. The inclusion criteria were as follows: (1) studies reporting on the efficacy or safety of natural products or small-synthetic-molecule drugs in NAFLD, (2) articles discussing NAFLD pathogenesis or therapeutic mechanisms, and (3) clinical trials, preclinical studies, and high-quality reviews. The exclusion criteria included irrelevant articles, case reports, and studies lacking sufficient data. All included studies were critically evaluated for methodological quality and relevance to ensure the robustness of the review.

## 3. Classification and Therapeutic Advances of Natural Products

Natural products have a wide range of chemical structures and biological activities. Based on their chemical properties and mechanisms of action, natural products can be categorized into flavonoids, terpenoids, saponins, polyphenols, alkaloids, polysaccharides, and other compounds. These substances exert their therapeutic effects through different pathways, including metabolic modulation, anti-inflammatory and antioxidant properties, and the modulation of the gut microbiota. Flavonoids have strong anti-inflammatory, antioxidant, and lipid-modulating properties [20]. Numerous studies have emphasized the potential of flavonoids in alleviating NAFLD through mechanisms such as enhancing lipid metabolism, reducing oxidative stress, and modulating inflammatory pathways [21]. Similarly, terpenoids have emerged as promising agents for metabolic disorders, with specific compounds like betulinic acid, ursolic acid, and glycyrrhizic acid demonstrating effectiveness in regulating lipid metabolism. Saponins also contribute to lipid metabolism regulation, inflammation modulation, and the maintenance of gut microbiota balance. Polyphenols, recognized for their antioxidative and anti-inflammatory properties, further offer therapeutic potential against NAFLD through mechanisms that improve lipid metabolism and support gut health. Alkaloids are notable for their role in modulating the gut microbiota and reducing hepatic lipid deposition, while polysaccharides contribute to NAFLD management through their ability to enhance intestinal barrier function and modulate immune responses. Not only these categories, but also other natural products play an important role. The following will be elaborated in sequence.

### 3.1. Flavonoids

#### 3.1.1. Nobiletin (NOB)

NOB, a citrus flavonoid, alleviates hepatic steatosis mainly through transcription factor EB (TFEB)-mediated lysosomal biogenesis and lipophagy [22]. Studies have demonstrated that NOB alleviates lipid accumulation, oxidative stress, and inflammatory responses in NAFLD by regulating key signaling pathways, including Nrf2, SREBP-1c, and NF-κB [23]. In addition, NOB reverses gut microbiota dysbiosis and attenuated hepatic lipid accumulation under metabolic stress in NAFLD mice [24]. NOB significantly increases plasma adiponectin levels, upregulated hepatic adiponectin receptor 1 (AdipoR1) expression, and downregulated the expression of Srebp1c, Acaca1, Tnfα, and Fgf21 in the liver, while lowering serum insulin levels. These results indicate that NOB can reduce hepatic lipid accumulation and attenuate steatosis in db/db mice [25,26]. Furthermore, NOB not only inhibits lipid biosynthesis, but also promotes lipid catabolism, thereby ameliorating hepatic steatosis and oxidative stress [27].

#### 3.1.2. Quercetin (QUE)

QUE, a flavonoid with strong antioxidant properties, significantly reduces lipid accumulation in adipocytes and decreases the expression of SREBP-1 and XBP-1 and their fatty acid synthesis gene targets. QUE directly exerts anti-adipogenic effects by inhibiting the de novo lipogenesis (DNL) pathway through the ACACA/AMPK/PP2A axis [28]. A randomized controlled trial found that quercetin treatment for 12 weeks significantly reduced the liver lipid content in patients with NAFLD [29]. Extensive pharmacological studies suggest that quercetin alleviates NAFLD through AMPK-mediated mitophagy, improving inflammation, oxidative stress, and lipid metabolism [30,31]. Furthermore, quercetin can mitigate high-fat diet (HFD)-induced hepatic lipotoxicity by targeting mitochondrial ROS-mediated ferroptosis [32]. For T2DM-related NAFLD, quercetin regulates cholesterol homeostasis and converts cholesterol into bile acids (BAs) by downregulating the mTOR/YY1 signaling pathway, exerting hepatoprotective effects [33]. Quercetin also prevents liver steatosis and fibrosis by downregulating the miR-21 transcription and upregulating the Nrf2 expression [34].

#### 3.1.3. Naringenin

Naringenin, a flavonoid derived from citrus fruits, regulates various biological processes associated with NAFLD [35]. Supplementation with naringenin may be beneficial for cardiovascular complications in NAFLD patients [36]. Naringenin improves lipid metabolism in rats by increasing energy expenditure and modulating autophagy via the direct or indirect activation of AMPK [37]. Recent studies have indicated that the therapeutic effects of naringenin on NASH are associated with the activation of liver SIRT1-mediated signaling cascades [38].

#### 3.1.4. Luteolin

Luteolin is a natural plant flavonoid with anti-lipogenic properties that inhibits the synthesis of phospholipids and cholesterol [39]. Luteolin lowers serum triglycerides, total cholesterol, alanine aminotransferase (ALT), and low-density lipoprotein cholesterol (LDL-C) levels while enhancing mitochondrial enzyme activity, including succinate dehydrogenase. Luteolin promotes mitochondrial biogenesis via the AMPK/PGC-1α pathway [40]. Studies have found that luteolin, in combination with zinc oxide (ZnO) nanoparticles, may alleviate NAFLD by reducing insulin resistance, improving oxidative stress, and modulating insulin signaling [41]. Moreover, luteolin inhibits TLR4 signaling in the liver, reducing the secretion of pro-inflammatory factors, while restoring and repairing damaged intestinal mucosal barriers and the gut microbiota imbalance [42].

#### 3.1.5. Taxifolin

Taxifolin (also known as dihydroquercetin) is a natural flavonoid with antioxidant and anti-inflammatory properties [43]. Studies have shown that taxifolin exhibits excellent activity in preventing and treating liver damage [44]. In NASH mouse models, taxifolin significantly prevented hepatic steatosis, chronic inflammation, and liver fibrosis [45]. Its mechanisms include direct action on hepatocytes to inhibit lipid accumulation, enhancing brown adipose tissue activity and suppressing weight gain. Taxifolin also alleviates inflammatory responses induced by caspase-1 activation in steatotic hepatocytes, inhibiting lipid accumulation and caspase-1-associated pyroptosis, and demonstrates therapeutic effects on alcohol- and HFD-induced fatty liver [46].

#### 3.1.6. Silymarin

Silymarin, derived from the plant Silybum marianum, is a flavonoid compound with multiple hepatoprotective properties, including antioxidant and lipid-lowering effects, although its bioavailability is low [47]. The main active ingredient in silymarin is silybin which can reduce lipid accumulation by activating peroxisome proliferator-activated receptor (PPAR)-α [48,49]. Research indicates that silymarin significantly improves NAFLD, as evidenced by the reduced hepatic lipid droplet accumulation, enhanced liver function, decreased hepatic triglycerides (TG) and serum total cholesterol (TC), and restored glucose tolerance [50]. Moreover, silymarin supplementation improves insulin resistance and reduces hepatic levels of inflammatory cytokines TNF-α and IL-6 [51].

#### 3.1.7. Icariin (ICA)

ICA, a flavonoid compound derived from the herb Epimedium, exhibits ferroptosis inhibitory activity. Studies indicate that ICA mitigates NASH by suppressing ferroptosis through the modulation of the Nrf2-xCT/GPX4 pathway [52]. Notably, rats treated with ICA show significantly higher plasma concentrations of icariin’s major metabolite, icaritin (ICT), which, in turn, exerts anti-adipogenic effects associated with reduced endoplasmic reticulum (ER) stress [53]. ICA improves fatty acid oxidation and reduces hepatic lipid accumulation [54]. Additionally, ICA alleviates NAFLD by upregulating miR-206, which mediates the NF-κB and MAPK signaling pathways [55]. Other studies suggest that ICA may also act through the activation of the AMPKα1/PGC-1α/GLUT4 pathway [56].

#### 3.1.8. Caffeic Acid Phenethyl Ester (CAPE)

CAPE, a natural flavonoid compound found in propolis, is an inhibitor of bacterial bile salt hydrolase (BSH) [57,58]. CAPE improves obesity-related steatosis, at least in part, by inhibiting BSH activity through the gut microbiota–bile acid–farnesoid X receptor (FXR) pathway [59]. CAPE also improves metabolism by inducing PPAR-γ activation and remodeling adipose tissue [60].

### 3.2. Terpenoids

#### 3.2.1. Betulinic Acid

Betulinic acid, a pentacyclic triterpenoid widely found in birch and other plants, is a novel FXR agonist [61]. The activation of FXR by betulinic acid reduces the lipotoxicity, enhances cholesterol excretion, and alleviates endoplasmic reticulum stress, thereby mitigating NAFLD [62]. Moreover, it effectively improves hepatic lipid accumulation by regulating the AMPK-mTOR-SREBP signaling pathway and protects hepatocytes from abnormal lipid deposition via the YY1/FAS pathway [63,64].

#### 3.2.2. Ursolic Acid

Ursolic acid, another widely distributed pentacyclic triterpenoid, is commonly found in various traditional herbal medicines [65]. As a novel liver X receptor α (LXRα) antagonist, it inhibits lipogenesis in NAFLD [66]. Ursolic acid also ameliorates high-fat-diet-induced hepatic steatosis by modulating key enzymes of lipid metabolism and the PPAR-α pathway [67]. Furthermore, it reverses hepatic fibrosis by inhibiting the NOX4/NLRP3 inflammasome pathway and gut microbiota dysbiosis [68]. It also modulates the IGF-IR and HIF-1 signaling pathways through proteoglycan-mediated mechanisms, thereby alleviating metabolic dysfunction and hepatic hypoxia [69].

#### 3.2.3. Glycyrrhizic Acid

Glycyrrhizic acid is an important triterpenoid that has been shown to improve NAFLD by modulating the gut microbiota. Studies have shown that glycyrrhizic acid significantly alters the composition of the gut microbiota and reduces carbohydrate transport and metabolic functions, thereby reducing hepatic fat accumulation. However, the exact molecular mechanism of glycyrrhizic acid’s action requires further study [70].

### 3.3. Saponins

#### 3.3.1. Gypenosides (GPs)

GPs, derived from Gynostemma pentaphyllum, alleviate hepatic steatosis and gut barrier damage in HFD-induced NAFLD models by activating the AMPK and TLR4/NF-κB pathways [71]. GPs regulate the expression of key genes involved in hepatic lipid metabolism, reducing fatty acid synthesis while promoting their transport and degradation [72]. Notably, GPs ameliorate NASH via the activation of FXR and FXR-dependent signaling pathways [72]. Among its active components, GPXIII effectively inhibits lipid accumulation and peroxidation in hepatocytes, significantly increasing the phosphorylation of SIRT1 and AMPK, which reduces acetyl-CoA carboxylase (ACC) activity. In NASH animal models, GPXIII markedly decreases the size and number of lipid droplets while attenuating liver fibrosis and inflammation [73].

#### 3.3.2. Ginsenosides

Ginsenosides, a group of triterpenoid saponins with hormone-like activities, exhibit broad anti-inflammatory and metabolic regulatory effects [74]. Ginsenoside F2 mitigates hepatic steatosis and macrophage inflammation by altering the LXRα co-regulator binding affinity [75]. Ginsenoside Rg1 suppresses inflammation, improves hepatic lipid accumulation, and alleviates liver injury in NAFLD models, potentially mediated through the regulation of Atf3 and Acox2 [76,77]. Furthermore, ginsenoside Rh4 alleviates hepatic steatosis, lobular inflammation, and bile acid dysregulation by modulating FXR signaling, while Rg5 exerts anti-NASH effects via the Notch1 pathway, which is critical for lipid metabolism and hepatocyte apoptosis prevention [78,79]. Ginsenoside Rc enhances SIRT6-dependent fatty acid oxidation and antioxidant activity via PPAR-α, providing protection against HFD-induced fatty liver disease [80]. Notably, ginsenoside Rk3 suppresses the PI3K/AKT pathway, ameliorating hepatic inflammation and modulating the gut microbiota, which highlights its potential for uncovering host–microbiome interactions [81].

#### 3.3.3. Diosgenin (DG)

DG, a natural SIRT1 activator, alleviates NAFLD through the SIRT1/PGC-1α pathway and mitigates fatty acid uptake by regulating SIRT6-mediated mechanisms [82,83]. DG improves hepatic dysfunction by modulating cholesterol metabolism and inhibits NAFLD progression, including lipid accumulation, inflammation, and fibrosis. Structurally similar to cholesterol, DG downregulates CYP7A1 expression, affecting bile acid profiles in the liver and feces, including CDCA, CA, and TCA levels [84]. Additionally, DG modulates bile acid metabolism via the hepatic FXR-SHP and intestinal FXR-FGF15 pathways, suggesting its protective role against NASH through altering the Clostridium populations and bile acid dynamics [85,86]. DG also acts as a natural NLRP3 inhibitor, exerting therapeutic effects on NAFLD through the NLRP3 inflammasome-dependent signaling pathway [87].

#### 3.3.4. Soyasaponins

Soyasaponin monomers (SS-A1 and SS-Bb) exhibit anti-NASH effects by targeting the gut microbiota (GM). In NASH mouse models, SS-A1 and SS-Bb alleviate steatohepatitis and fibrosis, reduce serum ALT, AST, and LPS levels, and decrease hepatic TNF-α, IL-6, TG, and cholesterol content. They alter the bile acid composition in the liver, serum, and feces while activating FXR signaling in the liver and ileum and enhancing intestinal tight junction proteins such as occludin and ZO-1. However, the protective effects of SS-A1 and SS-Bb are abrogated following GM depletion, underscoring the critical role of the gut microbiota in their therapeutic mechanisms [88].

### 3.4. Polyphenols

#### 3.4.1. Epigallocatechin Gallate (EGCG)

EGCG, the most abundant polyphenol in green tea, has demonstrated anti-obesity, anti-inflammatory, and anti-diabetic effects [89]. Studies show that EGCG alleviates NAFLD by inhibiting the expression and activity of DPP4, reducing lipid accumulation, and restoring the dysregulated lipid metabolism [90]. In addition, EGCG promotes the growth of beneficial intestinal microorganisms (e.g., Lactobacillus and Vibrio desulfuricans), increases the content of short-chain fatty acids (SCFAs), and decreases the content of lipopolysaccharides (LPSs). By inhibiting the TLR4/NF-κB pathway and activating the Nrf2 pathway, EGCG enhances the intestinal barrier function and attenuates hepatic steatosis and inflammatory responses [91]. Furthermore, EGCG protects the liver from lipotoxicity by inhibiting mitochondrial reactive oxygen species (ROS)-mediated iron metabolism, a key process in the deterioration of NAFLD [92].

#### 3.4.2. Curcumin

Curcumin is a polyphenol extracted from the plant turmeric and has potent anti-inflammatory and antioxidant properties [93]. Animal studies have shown that curcumin increased the proportion of beneficial bacteria and decreased the levels of pro-inflammatory markers, thereby reducing intestinal oxidative stress and inflammation. Curcumin strengthens intestinal tight junctions, thereby improving the intestinal barrier integrity and reducing systemic inflammation [94]. Beyond its gut-mediated effects, curcumin exhibits therapeutic potential in metabolic diseases by lowering blood glucose and lipid levels and enhancing insulin sensitivity [95]. Another animal experiment found that curcumin could partially alleviate hepatic steatosis by enhancing mitochondrial function through SIRT3 [96]. The combination of curcumin and resveratrol exerts synergistic effects in alleviating NAFLD through the PI3K/AKT/mTOR and HIF-1 signaling pathways [18]. Moreover, the curcumin derivative Cur5-8 improves fatty liver disease by activating AMPK and regulating autophagy [97].

#### 3.4.3. Resveratrol

Resveratrol, a widely recognized natural polyphenol, has anti-inflammatory, antioxidative, and anti-tumor properties, along with emerging anti-obesity effects. It modulates the gut microbiota composition by increasing beneficial bacteria and reducing harmful species, thereby improving gut permeability and alleviating gut dysbiosis caused by a high-fat diet [98]. Resveratrol ameliorates NAFLD by upregulating the hepatic LDLr and SR-BI gene expression, significantly reducing tumor necrosis factor-α (TNF-α) levels, and activating the AMPK/SIRT1 and anti-inflammatory signaling pathways [99,100].

#### 3.4.4. Theabrownin (TB)

TB, a polyphenol found in dark tea, has shown protective effects against NAFLD and obesity. It increases hepatic fibroblast growth factor 21 (FGF21) levels and reduces the phosphorylation of mitogen-activated protein kinase p38 in mice fed a methionine-choline-deficient (MCD) diet, alleviating oxidative stress and fibrosis [101]. In HFD-induced mice, TB improves lipid profiles in the liver and serum, enhances SCFA levels in the gut, and modulates serotonin signaling pathways via gut microbiota regulation [102,103]. The combination of TB and Poria cocos polysaccharides (PCPs) further amplifies these beneficial effects, significantly reducing serum and hepatic lipid levels [104].

### 3.5. Alkaloids

#### 3.5.1. Berberine (BBR)

BBR, a major active ingredient extracted from Berberis vulgaris, is known for its hypoglycemic and hypolipidemic properties, as well as its ability to ameliorate insulin resistance and modulate the gut microbiota [105]. These properties have made BBR a compound that has been extensively studied in the treatment of NAFLD, T2DM, and hyperlipidemia [106]. Mechanistically, BBR inhibits mitochondrial complex I in the gut and liver and suppresses lipid synthesis, thereby alleviating obesity and hepatic steatosis [107]. In addition, it modulates the endoplasmic reticulum (ER)-stress-induced ERK1/2 activation in macrophages and hepatocytes, thereby inhibiting PA/LPS-induced inflammatory responses [108]. A randomized controlled trial showed that BBR significantly reduced the hepatic lipid content, ameliorated hepatic inflammation, and attenuated hepatic injury in patients with NAFLD, demonstrating its wide range of metabolic activities [109].

#### 3.5.2. Mulberry Twig Alkaloids (SZ-As)

SZ-As have shown remarkable efficacy in ameliorating metabolic disturbances in HFD-induced obese mice. SZ-As effectively reduce body weight, fat mass, serum total cholesterol, and low-density lipoprotein levels. Intriguingly, SZ-As also modulate the gut microbiota composition and alter fecal metabolites in obese mice. Furthermore, SZ-As significantly increase goblet cell numbers while reducing inflammation-induced colonic damage and pro-inflammatory macrophage infiltration in HFD-fed mice, demonstrating its protective effects against diet-induced colonic inflammation [110]. SZ-As can also improve lipid metabolism, inhibit oxidative stress, and delay liver fibrosis by regulating the expression of PGC1 α and the KEAP1/NRF2 pathway [111].

#### 3.5.3. Neferine (NEF)

NEF, a natural alkaloid extracted from Nelumbinis plumula, exhibits diverse pharmacological properties, particularly in treating metabolic disorders. In vitro studies have revealed that NEF enhances AMPK and ACC phosphorylation in OA-stimulated HepG2 cells, suppressing lipid synthesis. NEF also reduces inflammation and fibrosis in LPS-stimulated HepG2 and LX-2 cells. In a mouse model of NASH, NEF treatment alleviates hepatic lipid deposition, inflammatory cell infiltration, and fibrosis, demonstrating its significant hepatoprotective effects [112].

### 3.6. Polysaccharides

#### 3.6.1. Yellow Tea Polysaccharide (YTP)

YTP is a natural biomolecule with significant therapeutic effects on various metabolic diseases. It has been shown that YTP modulates bile-salt-hydrolase-associated microbiota and activates the bile acid synthesis pathway, thereby inhibiting the progression of NAFLD [113]. It enhances the physical barrier function of the gut by maintaining mucosal integrity and promoting the production of tight junction proteins [114]. It also strengthens the intestinal immune barrier by suppressing pro-inflammatory immune cell subsets and cytokine profiles while promoting M2 macrophage polarization [115]. These properties collectively make YTP a promising agent for preventing and treating HFD-induced metabolic disorders [116].

#### 3.6.2. Pectic Polysaccharide YJ3A1

Pectic polysaccharide YJ3A1, a novel RG-I type polysaccharide purified from Rosa chinensis flowers, has demonstrated significant protective effects against NASH. The oral administration of YJ3A1 notably alleviates NASH-associated inflammation, oxidative stress, and fibrosis, without affecting the liver morphology in healthy mice at a dose of 50 mg/kg. Mechanistic studies reveal that the bioactivity of YJ3A1 may involve the inhibition of HMGB1 expression and release, thereby suppressing the HMGB1/TLR4/NF-κB and Akt signaling pathways to mitigate the progression of NASH [117].

### 3.7. Others

#### 3.7.1. Capsaicin

Capsaicin, a lipophilic compound and the primary active component of chili peppers, acts as a high-affinity agonist of transient receptor potential vanilloid subtype 1 (TRPV1) [118]. Dietary capsaicin has been shown to alleviate hepatic steatosis and insulin resistance in obese mice fed an HFD [119]. The underlying mechanisms may involve the upregulation of carnitine palmitoyltransferase 1 (CPT-1) and CD36 expression, promoting hepatic β-oxidation and a fatty acid influx. Concurrently, capsaicin reduces the expression of key enzymes involved in DNL, such as ACC and fatty acid synthase (FASN), thereby inhibiting lipid accumulation in the liver. These findings suggest that capsaicin enhances the lipid metabolic balance and offers a potential therapeutic strategy for NAFLD [120].

#### 3.7.2. Fucoxanthin

Fucoxanthin, a carotenoid mainly found in brown algae, exhibits multiple biological activities against NAFLD, including modulating the lipid metabolism, exerting anti-inflammatory and antioxidative effects, and demonstrating anti-fibrotic potential in preventing the progression of NASH [121]. Recently, a liver-targeting vesicle system (GA-LpEVs-FX) encapsulating fucoxanthin was developed, with a glycyrrhetinic acid (GA) modification via an amide reaction to enhance liver-specific delivery. In vitro studies demonstrated that GA-LpEVs-FX effectively reduced hepatic lipid accumulation and mitigated reactive oxygen species (ROS)-induced damage, as well as downregulated lipogenesis-related protein expression. In vivo, GA-LpEVs-FX exhibited a significant downregulation of adipogenesis-related proteins [122]. Fucoxanthin also regulated the lipid metabolism, oxidative stress, and inflammation via the AMPK/Nrf2/TLR4 signaling pathway and attenuated free fatty acid-induced NAFLD [123]. It not only inhibited hepatic oxidative stress and inflammation, but also prevented early fibrosis in diet-induced NASH model mice [123]. These results highlight the therapeutic potential of fucoxanthin and its formulations for NAFLD and NASH treatment.

#### 3.7.3. Emodin

Emodin (EMO), a naturally occurring anthraquinone derivative, is widely present in traditional herbal medicines [124]. Modern studies reveal that EMO improves NAFLD by reducing lipid droplet accumulation via the inhibition of the phosphorylated P38/P38 and phosphorylated ERK1/2 expression [125]. Furthermore, EMO alleviates HFD-induced lipid accumulation, insulin resistance, inflammation, and oxidative stress in a dose-dependent manner by suppressing FXR expression [126]. The therapeutic effects of EMO are closely associated with the regulation of the ERS-SREBP1c pathway and the activation of the CaMKK-AMPK-mTOR-p70S6K-SREBP1 signaling pathway, which significantly ameliorate hepatic steatosis [127,128].

#### 3.7.4. β-Sitosterol

β-sitosterol, one of the most abundant plant sterols, exhibits anti-obesity, anti-inflammatory, and anti-diabetic properties. Its antioxidant capacity and ability to promote pancreatic β-cell regeneration contribute to its anti-diabetic benefits [129]. β-sitosterol alleviates hepatic steatosis induced by HFD in rats and decreases serum triglycerides, aminotransferases (ALT and AST), and inflammatory markers. It also modulates protein-folding homeostasis by mitigating endoplasmic reticulum (ER) stress, evidenced by the reduced overexpression of IRE-1α, sXBP1, and CHOP. These findings underscore the multifaceted metabolic regulatory effects of β-sitosterol in protecting against NAFLD [130].

#### 3.7.5. Tanshinone IIA

Tanshinone IIA (TIIA), a diterpenoid extracted from Salvia miltiorrhiza, has been widely used to treat cardiovascular and liver diseases due to its potent anti-inflammatory and antioxidant activities. TIIA negatively regulates the ER-stress-induced unfolded protein response (UPR) through the activation of the PPAR-α/FGF21 axis, thereby alleviating the progression of NASH [131]. In addition, TIIA protects palmitate-exposed HepG2 cells by attenuating the excessive ER stress, ER-stress-induced apoptosis, and hepatic steatosis [132]. In HFD-fed mice, TIIA attenuates hepatic steatosis, restores the serum lipid distribution and glucose tolerance, and attenuates NASH-associated fibrosis by modulating the TGF-β1/Smad signaling pathway [133,134]. Moreover, TIIA is an ideal candidate for the treatment of NAFLD because it can inhibit lipogenesis and lipid accumulation by regulating the LXRα/SREBP1 pathway and target PPAR-γ and TLR4 to improve lipid metabolism and oxidative stress [135,136].

#### 3.7.6. Cinnamic Acid (CA)

CA and its isomer trans-cinnamic acid (TCA) exhibit therapeutic potential against NAFLD. CA mitigates hepatic lipid accumulation by inhibiting the lipogenesis and fatty acid uptake while enhancing fatty acid oxidation [137]. Notably, TCA demonstrates more pronounced effects in reducing liver enzymes (ALT and AST), pro-inflammatory markers (e.g., TNF-α), and lipid biomarkers [138]. The combined use of CA and TCA or their further optimization may provide innovative therapeutic strategies for NAFLD.

Some natural products for treating NAFLD are shown in Table 1.

## 4. Classification and Research Progress of Small-Synthetic-Molecule Drugs

### 4.1. Hepatic Lipid Metabolism

The liver plays a central role in lipid metabolism, and its dysfunction can lead to abnormal lipid accumulation, resulting in fatty liver disease, insulin resistance, and systemic metabolic disorders. Recent advances in the development of small-molecule drugs targeting the hepatic lipid metabolism have demonstrated significant potential. These drugs act on various metabolic targets, including LXRα, ACC, diacylglycerol O-acyltransferase 2 (DGAT2), FASN, FXR, and fibroblast growth factors (FGFs), to regulate lipid synthesis, breakdown, and transport, thereby improving metabolic abnormalities associated with fatty liver disease.

#### 4.1.1. LXRα Inhibitor

Oltipraz

Oltipraz, a synthetic dithiolethione, mitigates hepatic steatosis by inhibiting LXRα activity. A clinical trial showed that a 24-week treatment with Oltipraz significantly reduced the liver fat content in NAFLD patients [139]. In another animal study, the oral administration of Oltipraz attenuated liver inflammation and fibrosis in Nrf2-deficient mice fed an HFD, indicating that Oltipraz ameliorates liver damage in an Nrf2-independent manner [140].

#### 4.1.2. ACC Inhibitors

Firsocostat (GS-0976)

Firsocostat is an orally bioavailable ACC inhibitor targeting the liver, designed for treating NAFLD. Studies have shown that Firsocostat can be co-administered with CYP3A and UGT inhibitors without a dose adjustment, though it should not be used alongside potent OATP1B/3 inhibitors such as rifampicin or cyclosporine A [141]. Twelve weeks of treatment with 20 mg Firsocostat reduced hepatic steatosis and fibrosis [142].

Clesacostat (PF-05221304)

Clesacostat has shown efficacy in reducing hepatic steatosis, inflammation, and serum triglyceride levels [143]. The combination of Clesacostat with Ervogastat demonstrated a 36% incidence of adverse events (AEs), none of which led to discontinuation. This combination mitigated the serum triglyceride elevation induced by ACC inhibition, addressing some limitations of monotherapy [144].

#### 4.1.3. DGAT2 Inhibitor

Ervogastat (PF-06865571)

DGAT2 is a key enzyme in TG synthesis [145]. Ervogastat, developed by Pfizer, is a DGAT2 inhibitor for NASH treatment [146,147]. DGAT2 inhibition prevents fatty acid storage as TG, counteracting the hyperlipidemia induced by ACC inhibitors [148].

#### 4.1.4. Stearoyl-CoA Desaturase-1 (SCD-1) Inhibitor

Aramchol

Aramchol, a partial inhibitor of SCD-1, has demonstrated improvement in hepatic steatosis and fibrosis in rodents [149]. In a Phase 2b trial, although the primary endpoint of a significant hepatic fat reduction with 600 mg aramchol was not met, its safety profile and observed changes in liver histology and biomarkers remain promising for further investigation [150].

#### 4.1.5. FASN Inhibitors

FT-4101

DNL is a fundamental biosynthetic pathway integrated into energy metabolism, particularly in insulin-resistant states, providing fatty acids for storage and circulation [151,152]. FT-4101, a FANS inhibitor, at 3 mg effectively reduced hepatic DNL and steatosis in NAFLD patients without major safety concerns [153].

TVB-2640

TVB-2640, a FASN inhibitor, targets excessive hepatic fat and lipotoxicity to suppress inflammation and fibrosis pathways. A 12-week trial showed dose-dependent reductions in hepatic fat and improvements in biochemical, inflammatory, and fibrosis markers in NASH patients [154].

#### 4.1.6. FXR Agonists

Obeticholic acid (OCA)

OCA is an FXR agonist that has been shown to increase total cholesterol and LDL-C in patients with non-alcoholic steatohepatitis (NASH). Although OCA improves liver disease, it may lead to an increase in cholesterol levels. However, the effect of OCA on cholesterol remains unclear. In recent studies, we observed that OCA treatment was associated with an undesirable increase in lipoprotein levels, which improved upon discontinuation of the treatment [155]. Treatment with OCA 25 mg significantly improved fibrosis and key components of NASH disease activity. The interim analysis of the ongoing trial showed significant histological improvement, suggesting potential clinical benefit [156]. NASH patients undergoing regeneration assessments exhibited an impaired quality of life at baseline, accompanied by potential pruritus. The observed improvements in NASH were aligned with improvements in several health-related quality of life (HRQoL) domains. Generally, mild pruritus was observed early during OCA treatment and did not worsen over time [157].

Cilofexor

Cilofexor is a non-steroidal FXR agonist that was well-tolerated in NASH patients over a 24-week treatment period. It significantly reduced liver fat, liver biochemistry, and serum bile acids [158]. Pruritus was reported in 20–29% of cilofexor-treated patients, compared to 15% in placebo-treated individuals [159]. Cilofexor is being evaluated for the treatment of NASH and primary sclerosing cholangitis (PSC). It increases plasma levels of FGF19 and dose-dependently reduces the serum bile acid intermediate 7α-hydroxy-4-cholestene-3-one (C4). Doses above 30 mg appear to activate FXR in the intestine. Cilofexor is generally well-tolerated, with all treatment-emergent AEs being mild or moderate, with headaches being the most common TEAE [160].

PX-104

PX-104 is an oral, non-steroidal FXR agonist and a key regulator of bile acid, glucose, and lipid homeostasis. After 4 weeks of treatment in non-diabetic NAFLD patients, PX-104 improved insulin sensitivity and liver enzymes. Further investigation is needed to explore its effects on fecal bile acids (BAs) and the gut microbiome [161].

TQA3526

TQA3526 is a novel FXR agonist being investigated for the treatment of NASH and primary biliary cholangitis (PBC). In healthy Chinese subjects, TQA3526 (< 10 mg/day) was found to be safe and well-tolerated. Its safety and pharmacokinetics/pharmacodynamics (PK/PD) characteristics support further evaluation in NASH or PBC patients [162].

Vonafexor

Vonafexor is a second-generation non-bile acid FXR agonist being investigated in NASH patients with suspected fibrosis. The Phase IIa LIVIFY trial results showed that the daily oral administration of vonafexor led to a reduction in liver fat, liver enzymes, fibrosis biomarkers, body weight, and waist circumference in the short term. It may also improve kidney function. Mild to moderate pruritus (a peripheral FXR effect) and LDL-C elevations were observed but could be managed with lower doses and statin therapy [163].

MET409

MET409 is an FXR agonist with a unique chemical structure that significantly reduced liver fat in NASH patients after 12 weeks of treatment, without significant AEs. These results provide the first clinical evidence suggesting that the risk–benefit profile of FXR agonists may be enhanced [164].

Tropifexor (TXR)

TXR led to sustained reductions in ALT and the liver fat fraction up to week 48. However, no similar trend was observed for AST by week 12. As with other FXR agonists, pruritus was commonly observed in a dose-dependent manner [165]. When combined with cenicriviroc (CVC), the safety of the TXR + CVC combination was similar to that of each drug alone, with no new safety signals. TXR monotherapy resulted in persistent reductions in ALT and body weight. Compared to monotherapy, the TXR + CVC combination did not show significant improvements in ALT, body weight, or histological endpoints [166].

#### 4.1.7. Apical Sodium-Dependent Bile Acid Transporter (ASBT) Inhibitor

Volixibat

Volixibat is an inhibitor of ASBT, which is hypothesized to improve NASH by blocking bile acid reabsorption and stimulating bile acid production in the liver. Previous studies have shown that volixibat can lower cholesterol levels in the blood. However, the clinical trial results show that volixibat did not reduce liver fat or have any beneficial effects on liver injury. Therefore, volixibat may not be an effective treatment for patients with fatty liver disease [167].

#### 4.1.8. Fibroblast Growth Factor 19/21 (FGF19/21) Analogs

Aldafermin

Aldafermin is an engineered analog of FGF19, a gut hormone, with good tolerability. However, clinical studies have shown no significant dose-dependent response in improving fibrosis at least in one stage, and no deterioration of NASH [168]. Aldafermin works by inhibiting bile acid synthesis and regulating metabolic homeostasis, leading to a reduction in liver fat [169]. It has shown a trend toward fibrosis improvement. In patients with compensated NASH cirrhosis, a 3 mg dose of aldafermin significantly reduced liver fibrosis [170].

Efruxifermin

Preclinical and clinical data suggest that FGF21 has potential antifibrotic effects, improves metabolic status, and is a promising treatment for NASH. Efruxifermin is a long-acting Fc-FGF21 fusion protein. Treatment with efruxifermin reduced the liver fat fraction in patients with F1–F3 stage NASH and demonstrated an acceptable safety profile [171]. In patients with F2 or F3 fibrosis, efruxifermin improved liver fibrosis and resolved NASH over 24 weeks with good tolerability, supporting further evaluation in Phase 3 trials [172].

Pegbelfermin (BMS-986036)

Pegbelfermin is an FGF21 analog that has been administered subcutaneously in NASH patients. Treatment for 16 weeks generally showed good tolerability and a significantly reduced liver fat fraction [173]. A 12-week regimen did not significantly affect glycated hemoglobin (HbA1c) levels, but higher weekly doses and daily doses were associated with improved metabolic parameters and fibrosis biomarkers in patients with obesity and fatty liver tendencies, particularly those with T2DM [174]. Subsequent studies have highlighted Pegbelfermin’s potential metabolic effects in NASH patients. Clinical trial results indicate that Pegbelfermin reduces liver fat, improves liver injury, and enhances metabolic factors and biomarkers associated with fibrosis [175]. Liver biopsy evaluations showed a ≥1 point reduction in the fibrosis score without NASH deterioration or, conversely, NASH improvement without fibrosis worsening. Over 48 weeks of treatment, Pegbelfermin demonstrated good tolerability [176].

Pegozafermin (BIO89-100)

Pegozafermin is a long-acting glycosylated FGF21 analog used for the treatment of NASH and severe hypertriglyceridemia. Its efficacy and safety in non-cirrhotic NASH patients, confirmed by biopsy, are not fully established. The most common AEs associated with pegozafermin treatment were nausea and diarrhea. In a Phase 2b trial, pegozafermin treatment resulted in an improvement in fibrosis, supporting its progression into Phase 3 development [177]. This therapy was generally well-tolerated and was associated with clinically meaningful reductions in liver fat, liver function, and circulating lipid levels. A further evaluation of Pegozafermin in NASH patients is warranted [178].

NGM282

NGM282 is an engineered analog of FGF19. In a 12-week treatment study, it improved the histological features of NASH, reduced the non-alcoholic fatty liver disease activity score (NAS) and fibrosis score, and also improved non-invasive imaging and serum biomarkers [179].

BFKB8488A

BFKB8488A is a bispecific antibody targeting FGF receptor 1c and Klothoβ. It has demonstrated adequate tolerability in patients with T2DM or NAFLD. Treatment with BFKB8488A resulted in reductions in triglycerides, improvements in high-density lipoprotein (HDL) cholesterol, and a trend toward improved liver health markers. Notably, in NAFLD patients, there was a significant reduction in liver fat [180].

### 4.2. Inflammatory and Fibrotic

The study of anti-inflammatory and fibrotic drugs in NAFLD is of great significance, and their efficacy mainly lies in reducing hepatic inflammation and fibrosis, and also improving liver function and patient prognosis.

#### 4.2.1. Apoptosis Signal-Regulating Kinase 1 (ASK1) Inhibitor

Selonsertib

ASK1 plays a key role in hepatocyte injury, inflammation, and fibrosis in NASH. Selonsertib is a selective ASK1 inhibitor, aimed at improving fibrosis associated with NASH by targeting this pathway. However, in a 48-week monotherapy study, Selonsertib did not show significant antifibrotic effects in patients with bridging fibrosis or compensated cirrhosis due to NASH, and it was not able to reduce fibrosis in patients with advanced liver scarring [181].

#### 4.2.2. Caspase Inhibitor

Emricasan

Emricasan is a broad-spectrum caspase inhibitor that reduces excessive apoptosis and inflammation. It has been shown to the lower portal pressure and improve the synthetic function in carbon-tetrachloride-induced cirrhotic mice. Despite its potential, emricasan did not demonstrate efficacy in treating decompensated NASH cirrhosis. While earlier studies suggested some benefits, a larger-scale randomized trial found no reduction in decompensation events or improvement in liver function in patients with decompensated NASH cirrhosis [182,183]. Caspase-mediated apoptosis and inflammation induced by lipotoxicity are considered central drivers of liver damage in NAFLD, but emricasan failed to significantly reduce liver inflammation or fibrosis in NASH patients [184,185].

#### 4.2.3. CCR2/CCR5 Antagonist

Cenicriviroc (CVC)

CVC is an oral dual antagonist of chemokine receptors CCR2 and CCR5, which has shown antifibrotic potential in preclinical and Phase IIb studies of NASH [186]. However, in Phase III trials, CVC failed to demonstrate significant efficacy in treating liver fibrosis. Nevertheless, it was well-tolerated and safe in patients with NASH and liver fibrosis [187]. The most common treatment-related AEs included diarrhea (2.4%), abdominal pain, nausea, ALT and aspartate aminotransferase (AST) elevation, hypertriglyceridemia, myalgia, pruritus, and rash (each 1.2%). Most of these events were mild and non-life-threatening. No clinically significant changes in liver function, liver chemistry, or liver parameters were observed from baseline to study completion. Therefore, CVC at 150 mg once daily was found to have good tolerability in patients with NASH and stage 0–4 liver fibrosis [188].

#### 4.2.4. Phosphodiesterase (PDE) Inhibitor

Pentoxifylline (PTX)

PTX, a PDE inhibitor, reduces hepatic fat accumulation, improves lipid profiles, and ameliorates insulin resistance by inhibiting TNF-α synthesis [189]. Its mechanism involves competing with LPS for binding to TLR4, thereby suppressing the TLR4/MyD88/NF-κB signaling pathway [190]. PTX also enhances fatty acid β-oxidation and prevents NASH-associated liver tumorigenesis by reducing chronic liver inflammation and lipogenesis [191,192]. A randomized controlled trial found that PTX treatment is associated with a significant reduction in oxidized fatty acids in NASH patients [193].

#### 4.2.5. Novel Pan-Phosphodiesterase Inhibitor

ZSP1601

ZSP1601 is a pan-phosphodiesterase inhibitor. It has demonstrated good safety and tolerability, and has shown an effective improvement in the liver chemical composition, hepatic fat content, and fibrosis in patients with NAFLD [194]. Another trial suggests using a dosage regimen of 100 mg twice daily for subsequent clinical studies [195].

### 4.3. Insulin Resistance

Insulin resistance is one of the main causes of NAFLD. The effectiveness of these drugs is demonstrated by their ability to improve insulin sensitivity, reduce the accumulation of fat in the liver, and alleviate the inflammatory and fibrotic processes in the liver, thereby helping to reverse the progression of the disease and reduce the incidence of complications.

#### 4.3.1. 11β-Hydroxysteroid Dehydrogenase Type 1 (11β-HSD1) Inhibitors

J2H-1702

11β-HSD1 is an enzyme that converts cortisone to cortisol, playing a critical role in glucose metabolism and inflammation regulation. J2H-1702, a novel 11β-HSD1 inhibitor, has demonstrated potential in improving insulin sensitivity, reducing inflammation, and preventing non-alcoholic steatohepatitis (NASH) in preclinical models. At all doses, J2H-1702 significantly inhibited 11β-HSD1 activity compared to the placebo. The maximum inhibition was observed 12–24 h post-administration, with the effect lasting for up to one day. Systemic exposure is dose-dependent, and a single oral dose of 300 mg was well-tolerated, with only mild side effects such as diarrhea and dizziness reported [196].

AZD4017

AZD4017 is an 11β-HSD1 inhibitor that exists in human adipocytes [197]. Research shows that AZD4017 can block the hepatic conversion of (13) C-cortisone to (13) C-cortisol in all patients. In individuals with NASH and T2DM, AZD4017 improves hepatic steatosis compared to the placebo [198].

#### 4.3.2. Sodium-Glucose Cotransporter-2 (SGLT2) Inhibitors

Tofogliflozin

Tofogliflozin, an SGLT2 inhibitor, demonstrates excellent tolerability and significantly reduces the magnetic resonance imaging proton density fat fraction (MRI-PDFF) in patients with NAFLD and T2DM [199]. It offers potential therapeutic benefits by improving the liver energy metabolism, inflammation, and fibrosis in NAFLD patients [200].

Dapagliflozin

Dapagliflozin is also an SGLT2 inhibitor. It significantly reduces the liver fat content (LFC) and proton fat content (PFC) in T2DM patients with NAFLD [201]. It also improves serum ALT, TNF-α, and IL-6 levels. While dapagliflozin alleviates fibrosis in patients with significant hepatic fibrosis, its effects on steatosis or fibrosis may be linked to reductions in body weight or visceral fat [202].

Empagliflozin

Empagliflozin has shown good efficacy in reducing hepatic steatosis and may provide protection against hepatic insulin resistance. The early administration of SGLT2 inhibitors is considered beneficial in T2DM patients with NAFLD [203].

Ipragliflozin

Long-term treatment with ipragliflozin improves hepatic fibrosis. It may serve as an effective option for managing diabetes with concurrent NASH, while also addressing glycemic control and obesity [204].

Licogliflozin

A study found that licogliflozin, at a dose of 150 mg, reduces serum ALT levels in NASH patients without notable safety concerns. Further studies with longer durations and combination therapies targeting different mechanisms are warranted to confirm its potential as a NASH treatment [205].

#### 4.3.3. Glucagon-like Peptide-1 (GLP-1) Receptor Agonists

Cotadutide

GLP-1 receptor agonists are modified peptides resistant to dipeptidyl peptidase-4 degradation, mimicking endogenous GLP-1 effects. They regulate glucose levels, lipid metabolism, and various physiological functions by enhancing insulin secretion, suppressing glucagon production and hepatic glucose output, slowing gastric emptying, and reducing appetite [206,207]. Whether GLP-1 receptor agonists directly improve NASH or exert effects indirectly through weight loss, insulin sensitivity, and glycemic control remains under investigation [208]. Cotadutide, a dual agonist targeting GLP-1 and glucagon receptors, improves hepatic metabolic health by promoting glycogenolysis in the human liver [209].

Dulaglutide

As a standard treatment for T2DM, dulaglutide reduces LFC and improves γ-glutamyl transferase (GGT) levels in NAFLD patients. However, it shows no significant improvement in liver stiffness or serum AST and ALT levels, suggesting its role in the early treatment of NAFLD in T2DM patients [210].

Semaglutide

Semaglutide exhibits dose-dependent improvements in NASH resolution, with remission rates of 5%, 9%, and 13% at 0.1 mg, 0.2 mg, and 0.4 mg doses, respectively, compared to 1% with the placebo [211]. Phase 2 trials showed significant NASH resolution but no notable improvement in fibrosis staging between groups [212]. Semaglutide effectively reduces steatosis and enhances metabolic features [213]. Its combination with firsocostat and/or cilofexor is generally well-tolerated and significantly improves hepatic steatosis and biochemical parameters compared to semaglutide alone [214].

Survodutide

Survodutide, a dual GLP-1/glucagon receptor agonist, demonstrates favorable pharmacokinetics and safety profiles in patients with compensated or decompensated liver cirrhosis. It reduces LFC, fibrosis markers, and body weight, highlighting its potential for NASH treatment, especially in patients excluded from conventional trials [215].

Efocipegtrutide (HM15211)

Efocipegtrutide, a long-acting GLP-1/glucagon/gastric inhibitory polypeptide triple agonist, has shown promising efficacy and controllable toxicity in preclinical and Phase 1 studies for NASH. Adaptive study designs have optimized patient sampling for further clinical development [216].

Efinopegdutide

Efinopegdutide is a GLP-1/glucagon receptor co-agonist. A study suggests that efinopegdutide (10 mg weekly) reduces LFC more effectively than semaglutide, suggesting its potential as a promising NASH therapy [217].

#### 4.3.4. PPAR Agonists

Aleglitazar

PPAR agonists have demonstrated therapeutic potential for NAFLD. Aleglitazar is a PPAR-α/γ agonist which shows improvements in non-invasive tests assessing hepatic steatosis and fibrosis, further supporting the utility of the PPAR pathway in managing NAFLD [218].

Saroglitazar

Saroglitazar, a dual PPAR-α/γ agonist, shows promise in the treatment of NAFLD and NASH. It simultaneously activates PPAR-α and PPAR-γ, delivering dual benefits by improving both glucose and lipid metabolism [219]. Clinical studies have demonstrated that saroglitazar reduces ALT levels, improves LFC, insulin resistance, and atherogenic dyslipidemia in patients with NASH. Preclinical studies further revealed its ability to ameliorate NASH-related histological changes [220].

Lanifibranor

Lanifibranor, a pan-PPAR agonist, targets key pathways involved in the pathogenesis of NASH, including metabolism, inflammation, and fibrosis. In a Phase 2b clinical trial, patients receiving 1200 mg lanifibranor achieved a significant reduction in the Steatosis–Activity–Fibrosis (SAF) score by at least 2 points without worsening fibrosis compared to the placebo group. However, the lanifibranor group exhibited a higher incidence of AEs, such as diarrhea, nausea, peripheral edema, and weight gain. These findings warrant further investigation in Phase 3 trials [221].

#### 4.3.5. Selective PPAR-α Modulator (SPPARMα)

Pemafibrate

Pemafibrate, a SPPARMα, has shown potential in treating NASH. Preclinical studies demonstrated improved histological features of NASH, while clinical trials revealed a reduction in ALT levels and liver stiffness. Although pemafibrate did not significantly reduce the liver fat content, it holds promise as a candidate for combination therapy with agents targeting hepatic fat reduction [222].

### 4.4. Other Potential Therapeutic Pathways

#### 4.4.1. Thyroid Hormone Receptor (THR) Agonist

Resmetirom

Resmetirom, a THR-β agonist, promotes fatty acid uptake, oxidation, bile acid synthesis, and cholesterol metabolism. In Phase 2 trials, resmetirom significantly reduced the hepatic fat content without worsening fibrosis and improved patient quality of life [223,224]. Phase 3 studies demonstrated its efficacy in reducing fibrosis at daily doses of 80 mg and 100 mg [225]. Resmetirom has been approved by the FDA for clinical use, marking a milestone in NASH management.

#### 4.4.2. AMP-Activated Protein Kinase (AMPK) Activator

PXL770

AMPK, an energy sensor, plays a central role in lipid metabolism, inflammation, and insulin sensitivity. PXL770, a novel direct AMPK activator, was well-tolerated in clinical studies [226]. While it did not achieve the primary endpoint of improving the hepatic fat content, PXL770 showed potential in improving metabolic parameters, including DNL, glucose levels, and insulin sensitivity [227]. These findings highlight AMPK activation as a promising pharmacological target for T2DM and NAFLD.

#### 4.4.3. Ketohexokinase (KHK) Inhibitor

PF-06835919

PF-06835919, a first-in-class KHK inhibitor, has shown the potential to reverse metabolic disorders in preclinical and clinical studies [228]. Over 16 weeks of treatment, PF-06835919 is safe and well-tolerated, and reduces the whole-liver fat (WLF) in patients with NAFLD and T2DM [229]. These results suggest that KHK inhibition may provide a novel therapeutic approach for addressing NAFLD and insulin resistance [230].

#### 4.4.4. A3 Adenosine Receptor (A3AR) Agonist

Namodenoson

Namodenoson, an A3 adenosine receptor agonist, exhibited good tolerability, with no treatment-emergent severe AEs, drug interactions, hepatotoxicity, or mortality observed. Clinical studies indicated that the daily dosing of namodenoson at 25 mg effectively improved liver enzyme levels, demonstrating its safety and potential efficacy for NASH treatment [231].

#### 4.4.5. Cyclophilin Inhibitor

Rencofilstat (RCF)

RCF is a novel cyclophilin inhibitor under development for NASH and hepatocellular carcinoma [232]. In a study involving subjects with F2/F3 NASH, RCF demonstrated favorable safety and tolerability profiles over 28 days of treatment. Pharmacokinetics were not altered by the presence of NASH. Reductions in ALT, ProC3, and C6M levels suggest that prolonged RCF treatment may exert antifibrotic effects [233].

Some small-synthetic-molecule drugs for treating NAFLD are shown in Table 2.

## 5. Combination Therapy of Natural Products and Small-Synthetic-Molecule Drugs

**Figure 2:**
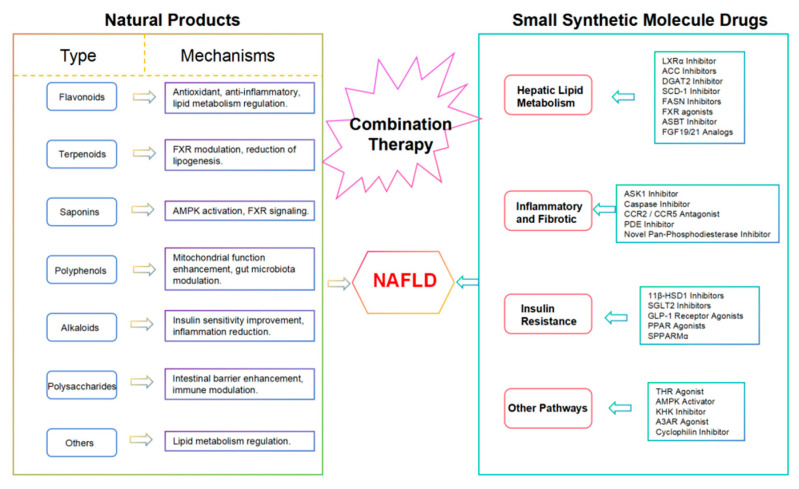
A mechanism flowchart of natural products and small-synthetic-molecule drugs.

Natural products and small-molecule drugs each offer unique advantages in the treatment of NAFLD. Combining these approaches can capitalize on their respective strengths and create synergistic effects. Natural products with multiple bioactive properties enhance overall health, while small-molecule drugs provide targeted and highly effective interventions. This combination therapy can also reduce the amounts of individual drugs used, thereby minimizing side effects and resistance. Although research on this strategy is currently limited, it has great potential to be explored for the future treatment of NAFLD. We have summarized a mechanism flowchart of natural products and small-synthetic-molecule drugs, as shown in Figure 2.

### 5.1. Complementary Effects

The mechanisms of action of natural products and small-molecule drugs can complement each other to intervene more comprehensively in the pathogenesis of NAFLD. For example, Cur5-8, a curcumin derivative, may ameliorate fatty liver by activating AMPK and autophagy regulation. Meanwhile, EW-7197 (vactosertib), a small-molecule inhibitor of TGF-β receptor I, reduces oxidative stress and fibrosis through the classical SMAD2/3 signaling pathway. The combined use of Cur5-8 and EW-7197 in NASH-induced mice and fibrotic hepatocytes attenuated hepatic fibrosis and steatohepatitis while preserving the benefits of both drugs [97]. Similarly, the anti-inflammatory and hepatoprotective drug bicyclol and the multi-targeted natural product BBR have shown beneficial effects on NAFLD in nutritive mouse models and patients. The combination of these two drugs not only reduced serum aminotransferase levels, hepatic triglycerides, and cholesterol, but also significantly attenuated steatosis, hepatic inflammation, and ballooning degeneration in NASH mice compared with monotherapy. Notably, bicyclol and BBR did not exhibit adverse interactions, but rather enhanced their overall therapeutic effects in improving NAFLD [234].

### 5.2. Synergistic Effects

Combination therapies can also yield synergistic benefits. For example, dapagliflozin and silymarin exhibited synergistic hepatoprotective effects in Wistar rats with carbon tetrachloride (CCl4)-induced toxicity by modulating the Nrf2 and HO-1 signaling pathways [235]. Another study evaluated the combination of metformin (MET), an oral hypoglycemic agent, and mallow extract (MAL), a natural product, in treating liver damage in HFD/STZ-induced diabetic rats. The combination therapy effectively alleviated hyperglycemia, insulin resistance, dyslipidemia, and NAFLD compared to either treatment alone. This evidence suggests that combining MET and MAL may represent a promising strategy with which to combat NAFLD in type 2 diabetic rats by improving lipid and glucose metabolism and suppressing inflammation [236].

### 5.3. Reduction in Side Effects

Combination therapy can also reduce the side effects associated with monotherapy. Natural products, known for their broad pharmacological activity and lower toxicity, can lower the required dosage and associated risks of small-molecule drugs when used together. For instance, resveratrol combined with half the dose of fenofibrate improved the NASH-related fructose-induced dysregulation of gene expression to a degree comparable to full-dose fenofibrate. This suggests that combination therapy could mitigate the risk of fenofibrate-related side effects [237]. Similarly, Schisandra chinensis extract demonstrated potential in preventing HFD-induced hepatic steatosis and enhancing the hepatic antioxidant status. When co-administered with atorvastatin, which has hepatotoxic potential, Schisandra extract significantly mitigated atorvastatin-induced liver toxicity and oxidative stress, while also benefiting weight control and lipid metabolism [238].

Some combination strategies for treating NAFLD are shown in Table 3.

### 5.4. Challenges and Future Directions

Translating the promising findings of combination therapy into real-world practice requires addressing critical aspects such as clinical relevance, safety profiles, patient adherence, and cost implications. While natural products exhibit potential efficacy, concerns about their safety persist, as certain herbal compounds may cause hepatotoxicity at high doses or with prolonged use. Similarly, small-synthetic-molecule drugs, such as FXR agonists, have been associated with side effects like gastrointestinal disturbances, pruritus, and altered lipid profiles. Patient adherence also remains a significant barrier; sustaining lifestyle interventions demands substantial dietary and behavioral changes, while long-term pharmacological treatments face challenges like medication fatigue, adverse effects, and complex dosing regimens. Simplified treatment protocols, coupled with enhanced patient education, could mitigate these issues. Furthermore, the cost of treatment poses a major challenge, with effective options like GLP-1 receptor agonists often being prohibitively expensive and limited in accessibility across regions due to geographic and regulatory disparities. Addressing these cost and accessibility barriers through cost-effectiveness evaluations and equitable distribution strategies is crucial.

Despite advances, significant gaps in understanding NAFLD treatment remain. The heterogeneity of NAFLD progression is poorly addressed, with a limited exploration of therapy effects on diverse patient subgroups differentiated by age, genetic predisposition, or comorbidities. Additionally, robust evidence regarding the long-term safety and efficacy of combination therapies, especially those involving natural products, is lacking. Innovations in research methodologies, including multi-omics approaches (e.g., genomics, metabolomics, and proteomics), offer opportunities to elucidate disease mechanisms and identify novel therapeutic targets. Given the multifactorial nature of NAFLD, interdisciplinary collaboration among clinicians, pharmacologists, and bioinformaticians is essential. International partnerships can enhance resource sharing, standardize research protocols, and support large-scale multicenter clinical trials. Beyond scientific challenges, regulatory frameworks and economic considerations play pivotal roles in advancing new therapies. Standardized evaluation criteria for the safety and efficacy of combination treatments, along with cost-effectiveness studies, are essential in order to ensure their feasibility in diverse healthcare contexts. Precision medicine approaches, guided by biomarkers predicting treatment responses, can further optimize therapeutic strategies, enhancing efficacy while minimizing side effects. Addressing these challenges through coordinated, multidisciplinary efforts will bridge current knowledge gaps and pave the way for safe, effective, and accessible therapies for NAFLD.

## 6. Current Status of Clinical Applications

A case highlights the therapeutic potential of silymarin in managing grade II NASH in a patient with type 2 diabetes and hyperlipidemia, where conventional treatments were contraindicated. Over two years, silymarin significantly improved liver enzymes (ALT: 299 to 72 U/L, and AST: 73 to 42 U/L) and lipid profiles without adverse effects, demonstrating its antioxidative, anti-inflammatory, and antifibrotic benefits. This suggests silymarin as a safe and effective adjunctive therapy for NAFLD management [239]. There is another case described: a 70-year-old overweight man with NAFLD showed significant improvement in liver enzyme levels after treatment with silymarin 140 mg twice daily for three months, demonstrating its potential as a safe and effective intervention for NAFLD management [240]. A 67-year-old woman with type 2 diabetes and NASH experienced significant clinical and histological improvements in liver function after four months of treatment with the SGLT2 inhibitor ipragliflozin, including normalized ALT and ferritin levels, reduced fibrosis markers, decreased liver fat, and improved steatosis, inflammation, and ballooning [241]. Another report on ipragliflozin describes a 56-year-old Japanese woman with slowly progressive type 1 diabetes mellitus (SPT1D), metabolic syndrome, and non-alcoholic fatty liver disease, exhibiting severe insulin resistance. Following treatment with ipragliflozin, the patient experienced significant improvements in insulin sensitivity, and reductions in blood glucose levels, visceral fat, and hepatic lipid content, as well as improvements in dyslipidemia. These findings suggest the potential efficacy of ipragliflozin therapy in managing obese SPT1D patients with insulin resistance [242].

## 7. Conclusions

The combination of natural products and small-synthetic-molecule drugs offers a promising approach to the treatment of NAFLD, utilizing their complementary mechanisms of action. Natural products can exert different biological activities against multiple pathways, while synthetic drugs can provide precisely targeted interventions. The combination of the two could improve efficacy, realize synergistic effects, and reduce the side effects associated with large doses of a single drug. However, rigorous preclinical and clinical studies are needed to optimize the combination and ensure safety and efficacy. This strategy has great potential to improve the treatment of NAFLD and bridge the gap between experimental research and clinical application.

## Figures and Tables

**Table 1:** Natural products for the treatment of NAFLD.

Type	Natural Products	Target	Mechanisms	Ref.
Flavonoids	Nobiletin	TFEB	Alleviating hepatic steatosis	[22]
		Nrf2, SREBP-1c, and NF-κB	Ameliorating lipid accumulation, oxidative stress, and inflammatory responses	[23]
		AdipoR1	Reducing hepatic lipid accumulation and attenuating steatosis	[25]
		Srebp1c, Acaca1, Tnfα, and Fgf21	Attenuating steatosis via downregulation of hepatic lipid accumulation	[26]
	Quercetin	Acetyl-CoA Carboxylase 1/AMPK/PP2A Axis	Reducing lipid accumulation in adipocytes	[28]
		AMPK	Improving inflammation, oxidative stress, and lipid metabolism	[30,31]
		mTOR/YY1	Regulating cholesterol homeostasis and converting cholesterol into bile acids to exert hepatoprotective effects	[33]
		miR-21 and Nrf2	Preventing liver steatosis and fibrosis	[34]
	Naringenin	AMPK	Improving lipid metabolism in rats by increasing energy expenditure and modulating autophagy	[37]
		SIRT1	Improving metabolic parameters and suppressing hepatic steatosis	[38]
	Luteolin	AMPK/PGC-1α	Increase mitochondrial biogenesis to ameliorate steatosis	[40]
		TLR4	Reducing the secretion of pro-inflammatory factors, while restoring and repairing damaged intestinal mucosal barriers and gut microbiota imbalance	[42]
	Taxifolin	Caspase-1	Reducing the inflammatory response induced by caspase-1 activation in steatotic liver cells and inhibit lipid accumulation	[46]
	Silymarin	PPARα	Reducing lipid accumulation	[49]
	Icariin	Nrf2-xCT/GPX4	Alleviating NASH by inhibiting ferroptosis	[52]
		NF-κB and MAPK	Improving fatty acid oxidation and reducing hepatic lipid accumulation	[55]
		AMPKα1/PGC-1α/GLUT4	Increasing insulin sensitivity and reducing lipid accumulation	[56]
	Caffeic Acid Phenethyl Ester	FXR	Improving obesity-related steatosis, at least in part, by inhibiting BSH activity	[59]
		PPAR-γ	Improving metabolism	[60]
Terpenoids	Betulinic Acid	FXR	Reducing lipotoxicity, enhancing cholesterol excretion, and alleviating endoplasmic reticulum stress	[62]
		AMPK-SREBP	Improving hepatic lipid accumulation	[63]
		YY1/FAS	Protecting hepatocytes from abnormal lipid deposition	[64]
	Ursolic Acid	LXRα	Inhibiting lipogenesis in NAFLD	[66]
		PPAR-α	Ameliorating high-fat-diet-induced hepatic steatosis	[67]
		NOX4/NLRP3	Reversing hepatic fibrosis	[68]
		IGF-IR and HIF-1	Alleviating metabolic dysfunction and hepatic hypoxia	[69]
	Glycyrrhizic Acid	Gut microbiota	Reducing carbohydrate transport and metabolic functions, thereby reducing hepatic fat accumulation	[70]
Saponins	Gypenosides	AMPK and TLR4/NF-κB	Alleviating hepatic steatosis and gut barrier damage in HFD-induced NAFLD models	[71]
		FXR	Attenuating liver fibrosis and inflammation	[72]
		SIRT1 and AMPK	Inhibiting lipid accumulation and peroxidation in hepatocytes	[73]
	Ginsenoside F2	LXRα	Mitigating hepatic steatosis and macrophage inflammation	[75]
	Ginsenoside Rg1	Atf3 and Acox2	Improving liver function and relieving pathological process	[77]
	Ginsenoside Rh4	FXR	Alleviating hepatic steatosis, lobular inflammation, and bile acid dysregulation	[78]
	Ginsenoside Rg5	Notch1	Improving lipid metabolism and hepatocyte apoptosis	[79]
	Ginsenoside Rc	PPAR-α	Enhancing SIRT6-dependent fatty acid oxidation and antioxidant activity	[80]
	Ginsenoside Rk3	PI3K/AKT	Ameliorating hepatic inflammation and modulating gut microbiota	[81]
	Diosgenin	SIRT1/PGC-1α	Improving mitochondrial dysfunction, fatty acid oxidation, lipid accumulation, steatosis, oxidative stress, and hepatocyte inflammation	[82]
		SIRT6	Mitigating fatty acid uptake	[83]
		CYP7A1	Improving hepatic dysfunction by modulating cholesterol metabolism	[84]
		FXR-SHP and FXR-FGF15	Modulating bile acid metabolism	[85,86]
		NLRP3	Attenuating lipid accumulation and liver injury	[87]
	Soyasaponins	FXR	Altering bile acid composition in the liver, serum, and feces	[88]
Polyphenols	Epigallocatechin Gallate	DPP4	Reducing lipid accumulation and restoring dysregulated lipid metabolism	[90]
		Mitochondrial ROS	Protecting the liver from lipotoxicity	[92]
	Curcumin	SIRT3	Partially alleviating hepatic steatosis by enhancing mitochondrial function	[96]
	Resveratrol	LDLr and SR-BI	Improved lipid concentration in serum and liver of rats	[99]
		AMPK/SIRT1	Improving liver function and reducing fatty liver indicators	[100]
	Theabrownin	FGF21 and p38	Alleviating oxidative stress and fibrosis	[101]
Alkaloids	Berberine	ERK1/2	Inhibiting PA/LPS-induced inflammatory responses	[108]
	Mulberry twig alkaloids (SZ-A)	KEAP1/NRF2	Improving lipid metabolism, inhibit oxidative stress	[111]
	Neferine (NEF)	AMPK	Suppressing lipid synthesis	[112]
Polysaccharides	Yellow Tea Polysaccharide	Bile acid metabolism	Inhibiting liver cholesterol accumulation in NAFLD mice	[113]
	Pectic Polysaccharide YJ3A1	HMGB1/TLR4/NF-κB and Akt	Alleviating NASH-associated inflammation, oxidative stress, and fibrosis	[117]
Others	Capsaicin	ACC and FASN	Inhibiting lipid accumulation in the liver	[120]
	Fucoxanthin	AMPK/Nrf2/TLR4	Relieving the level of oxidative stress and inflammation in hepatocytes	[123]
	Emodin	P38 and phosphorylated ERK1/2	Reducing lipid droplet accumulation.	[125]
		FXR	Improving lipid accumulation, insulin resistance, inflammation, and oxidative stress	[126]
		ERS-SREBP1c	Improving the lipid accumulation	[127]
		CaMKK-AMPK-mTOR-p70S6K-SREBP1	Ameliorating hepatic steatosis	[128]
	β-sitosterol	ER stress	Alleviating hepatic steatosis induced by HFD in rats and decreasing serum triglycerides, aminotransferases (ALT and AST), and inflammatory markers	[130]
	Tanshinone IIA	PPARα/FGF21	Negatively regulating the ER-stress-induced unfolded protein response	[131]
		TGF-β1/Smad	Attenuating hepatic steatosis, restoring serum lipid distribution and glucose tolerance, and attenuating NASH-associated fibrosis	[134]
		LXRα/SREBP1	Inhibiting lipogenesis and lipid accumulation	[135]
		PPAR-γ and TLR4	Improving lipid metabolism and oxidative stress	[136]
	Cinnamic Acid	ACLY, ACC, FAS, SCD1, PPAR-γ, CD36, CPT1A, PGC1α, and PPAR-α	Inhibiting hepatic lipogenesis and fatty acid intake, and increasing fatty acid oxidation	[137]

**Table 2:** Small-synthetic-molecules drugs for the treatment of NAFLD.

Therapeutic Targets	Classification	Small-Synthetic-Molecules Drugs	Clinical Trial Findings	Ref.
Hepatic Lipid Metabolism	LXRα Inhibitor	Oltipraz	Week treatment with oltipraz significantly reduced liver fat content in NAFLD patients	[139]
	ACC Inhibitors	Firsocostat (GS-0976)	12-weeks of treatment with 20 mg firsocostat reduced hepatic steatosis and fibrosis	[142]
		Clesacostat (PF-05221304)	Reducing hepatic steatosis, inflammation, and serum triglyceride levels	[143]
	DGAT2 Inhibitor	Ervogastat (PF-06865571)	Preventing fatty acid storage as TG	[147,148]
	SCD-1 Inhibitor	Aramchol	The primary endpoint of significant hepatic fat reduction with 600 mg aramchol was not met	[150]
	FASN Inhibitors	FT-4101	It was found that 3 mg effectively reduced hepatic DNL and steatosis in NAFLD patients without major safety concerns	[153]
		TVB-2640	Dose-dependent reductions in hepatic fat and improvements in biochemical, inflammatory, and fibrosis markers in NASH patients	[154]
	FXR Agonists	Obeticholic acid (OCA)	Treatment with OCA 25 mg significantly improved fibrosis and key components of NASH disease activity. Mild pruritus was observed early during OCA treatment and did not worsen over time	[156,157]
		Cilofexor	Reducing liver fat, liver biochemistry, and serum bile acids	[158]
		PX-104	Improving insulin sensitivity and liver enzymes	[161]
		TQA3526	Increasing circulating FGF-19 and decreasing C4 levels in a dose-dependent manner	[162]
		Vonafexor	Reducing liver fat, liver enzymes, fibrosis biomarkers, body weight, and waist circumference in the short term	[163]
		MET409	Significantly reducing liver fat in NASH patients after 12 weeks of treatment	[164]
		Tropifexor (TXR)	Leading to sustained reductions in ALT and liver fat fraction up to week 48	[165]
	ASBT Inhibitor	Volixibat	It cannot reduce liver fat and has no beneficial effect on liver damage	[167]
	FGF19/21 Analogs	Aldafermin	Inhibiting bile acid synthesis and regulating metabolic homeostasis, leading to a reduction in liver fat. A 3 mg dose of aldafermin significantly reduced liver fibrosis	[169,170]
		Efruxifermin	Reducing liver fat fraction in patients with F1–F3 stage NASH and demonstrated an acceptable safety profile. Improving liver fibrosis in patients with F2 or F3 fibrosis	[171,172]
		Pegbelfermin (BMS-986036)	Reducing liver fat, improves liver injury, and enhances metabolic factors and biomarkers associated with fibrosis	[175]
		Pegozafermin (BIO89-100)	Resulting in improvement in fibrosis	[177]
		NGM282	Improving histological features of NASH, reducing non-alcoholic fatty liver disease activity score (NAS) and fibrosis score, and also improving non-invasive imaging and serum biomarkers	[179]
		BFKB8488A	Reducing liver fat	[180]
Inflammatory and Fibrotic	ASK1 Inhibitor	Selonsertib	It has no significant anti fibrotic effect and cannot reduce fibrosis in patients with advanced liver scars	[181]
	Caspase Inhibitor	Emricasan	Failing to significantly reduce liver inflammation or fibrosis in NASH patients	[184,185]
	CCR2/CCR5 Antagonist	Cenicriviroc (CVC)	Failing to demonstrate significant efficacy in treating liver fibrosis	[187]
	PDE Inhibitor	Pentoxifylline (PTX)	Reducing lipid oxidation	[193]
	Novel Pan-Phosphodiesterase Inhibitor	ZSP1601	Improving liver chemical composition, hepatic fat content, and fibrosis in patients with NAFLD	[194]
Insulin Resistance	11β-HSD1 Inhibitors	J2H-1702	Systemic exposure is dose-dependent, and a single oral dose of 300 mg was well-tolerated, with only mild side effects such as diarrhea and dizziness reported	[196]
		AZD4017	Improving hepatic steatosis	[198]
	SGLT2 Inhibitors	Tofogliflozin	Improving liver energy metabolism, inflammation, and fibrosis in NAFLD patients	[200]
		Dapagliflozin	Reducing liver fat content (LFC) and proton fat content (PFC). Alleviating fibrosis	[201,202]
		Empagliflozin	Reducing hepatic steatosis and may provide protection against hepatic insulin resistance	[203]
		Ipragliflozin	Improving hepatic fibrosis	[204]
		Licogliflozin	Reducing serum ALT levels in NASH patients without notable safety concerns	[205]
	GLP-1 Receptor Agonists	Cotadutide	Promoting glycogenolysis in the human liver	[209]
		Dulaglutide	Reducing LFC and improving GGT levels in NAFLD patients	[210]
		Semaglutide	Reducing steatosis and enhancing metabolic features	[213]
		Survodutide	Reducing LFC, fibrosis markers, and body weight, highlighting its potential for NASH treatment	[215]
		Efocipegtrutide (HM15211)	Promising efficacy and controllable toxicity in preclinical and Phase 1 studies for NASH	[216]
		Efinopegdutide	Reducing LFC more effectively than semaglutide	[217]
	PPAR Agonists	Aleglitazar	Improving hepatic steatosis and fibrosis	[218]
		Saroglitazar	Reducing ALT levels, improving LFC, insulin resistance, and atherogenic dyslipidemia in patients with NASH	[220]
		Lanifibranor	Improving hepatic steatosis	[221]
	SPPARMα	Pemafibrate	Reducing ALT levels and liver stiffness	[222]
Other Potential Therapeutic Pathways	THR Agonist	Resmetirom	Reducing fibrosis at daily doses of 80 mg and 100 mg	[225]
	AMPK Activator	PXL770	Showing potential in improving metabolic parameters, including DNL, glucose levels, and insulin sensitivity	[227]
	KHK Inhibitor	PF-06835919	Reducing whole-liver fat (WLF) in patients with NAFLD and T2DM	[229]
	A3AR Agonist	Namodenoson	Daily dosing of namodenoson at 25 mg effectively improved liver enzyme levels	[231]
	Cyclophilin Inhibitor	Rencofilstat (RCF)	Reductions in ALT, ProC3, and C6M levels suggest that prolonged RCF treatment may exert significant antifibrotic effects	[233]

**Table 3:** Combination therapy of natural products and small-synthetic-molecules drugs for NAFLD.

Combined Effect	Natural Products	Small Synthetic Molecules	Mechanisms	Ref.
Complementary Effects	Cur5-8	EW-7197 (vactosertib)	Attenuating hepatic fibrosis and steatohepatitis while preserving the benefits of both drugs	[97]
	BBR	Bicyclol	Not only reducing serum aminotransferase levels, hepatic triglycerides, and cholesterol, but also significantly attenuating steatosis, hepatic inflammation, and ballooning degeneration in NASH mice compared with monotherapy	[234]
Synergistic Effects	Silymarin	Dapagliflozin	Exhibiting synergistic hepatoprotective effects in Wistar rats with carbon tetrachloride (CCl4)-induced toxicity	[235]
	MAL	MET	Improving lipid and glucose metabolism and suppressing inflammation	[236]
Reduction of Side Effects	Resveratrol	Fenofibrate	Mitigating the risk of fenofibrate-related side effects	[237]
	Schisandra chinensis extract	Atorvastatin	Mitigating atorvastatin-induced liver toxicity and oxidative stress, while also benefiting weight control and lipid metabolism	[238]

## Data Availability

Not applicable.
